# Book Reviews

**Published:** 1916-10

**Authors:** 


### BOOK REVIEWS

---

Books mentioned may be inspected at and ordered through this office. So far as possible, books received in any month will be reviewed in the issue of the second month following.

---

**National Parks Portfolio.** Issued by the Dept. of the Interior, Washington, D. C.

This consists of fasciculi of views of 9 of the great national parks. We advise our readers to apply for this collection of views. It is estimated that, last year, on account of the closing of Europe to tourists, $100,000,000 usually spent in

Europe, was put into circulation in the U. S. for quite as profitable recreation.

---

**Manual of Vital Function Testing Methods** and their Interpretation. Wilfred M. Barton, M. D., Washington. Published by Richard G. Badger, Boston. 255 pages, $1.50.

While, to some degree, tests bearing this name have been inserted in works on clinical diagnosis, never before have they been assembled and subjected to critical analysis in the aggregate. We make no attempt to define accurately this group of tests and to distinguish them from diagnostic tests in the aggregate, since the author has not done so. However the term is generally understood. The liver is discussed in regard to its glycobenetic, urea-forming, antitoxic, haemopoietic and exocrinous or biliary function—about 50 pages. Over 80 pages are devoted to the kidney and 30 to the pancreas. 35 pages are devoted to such cardiac and pulse tests as may be considered to represent functional power. Most of the rest of the book is devoted to the ductless glands. While the author makes no claim of original work, his previous experience in pharmacology and his more recent attention to clinical medicine in Georgetown University, have afforded opportunities for reviewing the subject critically and not merely as a compiler. It is noteworthy that this phase of diagnosis is very largely American, and that it is a topic in which science and practical application are joined to an unusual degree. The book is one of the rare instances in which there is almost nothing trite and in which one does not have to read many pages to get something unfamiliar and yet of practical value. Since the appearance of the first edition of Sicon's Clinical Diagnosis, we do not recall a book which is so radically novel and which, at the same time, fills a need of so large a part of the profession. It would be unjust to imply that equally original works, from the standpoint of authorship, have not appeared but, to the best of our recollection, they have either been monographs on some particular disease or method, or have dealt with subjects that are scarcely out of the theoretic stage, or have been limited in scope to certain specialties. Nor do we speak with derogation of systematic treatises which, in bringing a subject up to date, necessarily include much that is already familiar.

---

**Map of the State of N. Y. Showing Improved Highways** and Many Points of Historic Interest. Issued by the N. Y. State Commission of Highways, May 1, 1916, Edwin Duffey Commissioner, I. J. Morris, Sec'y.

Of the approximately 80,000 miles of roads, outside of cities and incorporated villages, 6,000 state and county highways have been improved by brick, cement, macadam or similar surfacing and 1,300 miles additional are under construction (not 13,000 as the disgruntled tourist might assume to be meant) besides 4,000 miles of road improved mainly by scraping and graveling by towns and counties. 6,500 miles of unimproved but commonly used and generally fairly good highways are also shown. This map is a boon to automobilists, among whom physicians are relatively if not actually, more numerous than members of any other business or profession except that of president (as of the country, railroads, banks and corporations).

There are approximately 250,000 automobiles licensed in the state, not to mention motor cycles. License fees, therefore, amount to nearly $2,000,000 a year. We understand that improved roads average something over $10,000 a mile, indicating that they are by no means paid for by owners of automobiles, as some of this class have imagined.

In no sense as a criticism of the Commission, but rather of the general subject, which is of importance not merely to tourists but to those using the roads for business purposes for all kinds of travel and commerce, and to the people as a whole both industrially and as a vital point in military preparedness, the following comment may be pardonable. The State is, approximately 300 miles wide and 100-150 miles from north to south, disregarding Long Island and the mountainous and scantily populated north-eastern extension. At first thought, 10,000 miles of improved highway would seem ample to provide for mutual accessibility of all important centers of population. Curiously enough, there is only one trunk route for the whole state, following approximately the old stage road to Albany and thence to New York. From this road, at Avon, a very sinuous highway reaches the principal cities of the southern tier. Nearly every city has a number of local roads available for a day's pleasure ride and there are a great many short connections between various pairs of cities or villages. It is true that, from almost any point to any other that would be naturally selected as a destination, there is a good deal of good road but the missing links, though often short in length are often sufficient in depth of mud or height of hills to be prohibitive except under favorable conditions. These conditions are satisfied too briefly for either business purposes or even the pleasure riding of a busy man. In the past, the demand for impartiality and the need of affording object lessons as to what a highway might become, required the pretty even distribution of road improvement but the time has arrived when even the interests of any particular locality as well as of the State as a whole, require concentration on missing links to secure trunk highways available at all times except those when the snow is deep—and when this has been accomplished, undoubtedly there will be a demand for snow plows.

We have already expressed the opinion that the macadam road is too short lived to be economic from the standpoint of taxation, either general or by license and that the sharp material and oil used in its construction and maintenance impose an indirect tax on rubber tired vehicles equal to the license tax. Thus, with some special exceptions, no further macadam roads should be laid, and those already constructed should be replaced with brick or cement as they wear out. For the immediate future, that is the next two or three years, we would advocate the building of brick or cement roads simply to connect short breaks in possible trunk highways. Two or three hundred miles of such construction would make nearly the whole 6,000 miles of state and county highways available for through travel in almost any direction, and would lengthen the radius of convenient travel from any average center, four or five times at least. Any additional funds, should, we believe, be devoted not to the construction of ideal roads but to rendering several times as much mileage of natural highways available for ordinary summer travel, by ordinary road work, consisting in grading dangerous hills, straightening out dangerous curves, scraping rough roads and filling in soft places with natural gravel, sand and shale.

---

**Medical Clinics of Chicago.** July, 1916, Vol. 2, No. 1. (W. B. Saunders Co., Phila., bi-monthly, $8 per year).

We are glad to note that James T. Case emphasizes the special value of fluoroscopy in the examination of the stomach. There is so much good matter that it is impossible to do justice to all the authors in a review.

The very clear and practical discussion of the anatomy and physiology of the spinal cord and its containers is aided by the large size of the pages, which allows illustrations of adequate size. Flatau's table of muscles in quoted with some modifications, giving the name of the muscles properly classified into regional groups, the spinal segment governing them and the functions. Bing's table of centers of skin and tendon reflexes is also quoted. Considerable space is devoted to general diagnostic methods, including the X-ray whose results are peculiarly effective in spina bifida. The various operations on the cord and its surrounding structures are described and depicted systematically. Indeed, while the author expresses conservative opinions as to operation in many instances, he does not attempt to cover medical and electric therapeusis and refers only briefly to electric diagnostic tests.

**Physics and Chemistry for Nurses.** Amy Elizabeth Pope, San Francisco, G. P. Putnam's Sons, New York (Knickerbocker Press), 444 pages, illustrated. $1.75.

This well known writer has shown singular skill in condensing various parts of the medical curriculum to meet the requirements of the trained nurse. While her critics, from different standpoints may not always agree as to the wisdom of teaching so much of the technical side of medicine to nurses or, on the other hand, may feel that in certain details or generally, any departure from the purely practical necessity of a training course should go to the limit of thoroughness,, we doubt whether any of these critics could undertake the task with better results. Whatever may be said as to restricting the training course to practical matters, her text books are certainly well adapted to the educated and ambitious trained nurse and to the laity who wish to obtain a fairly complete and intelligent understanding of medical sciences. We frankly admit, also, a personal appreciation of her books for a cursory review of subjects that have not been studied formally for a long time. In this book, the author has included a number of very practical applications of chemistry, as dyeing, food compositions, digestion, etc.

---

**A Manual of Otology. For Students and Practitioners.** By Charles Edwin Perkins, M. D., F. A. C. S., Professor of Clinical Otology in New York University and Bellevue Hospital Medical College; Associate Aural Surgeon to St. Luke's Hospital; Assistant Aural Surgeon, New York Eye and Ear Infirmary; Fellow American Otological Society, New York Otological Society, New York Academy of Medicine, etc. 12mo, 445 pages, with 120 engravings. Cloth, $3.00, net.

This is an excellent book, intermediate in size between the condensed and the very elaborate works. The author writes from long personal experience with the subject and has devoted especial attention to the internal ear and to the vexed question of the chords tympani, on which he offers a large amount of original research.

---

**Les Typhoides Intriquees.** Dr. Arthur Grimberg, published by Imprimerie de la Cour d'Appel, Paris. Paper cover, 64 pages.

The author has studied 160 cases from various Paris hospitals, working in the laboratory of Dr. A. Chantemesse. From the clinical side, special attention is given to false recurrences and recrudescences while the laboratory work deals with agglutinins and blood cultures, involving many technical improvements of an original nature.

---

**Treatment of Infantile Paralysis,** Robert W. Lovett, Boston. P. Blakiston's Son & Co., Philadelphia. 163 pages, 113 illustrations, $1.75.

It should be understood that this work does not attempt to discuss the treatment of the acute infection but rather speaks sceptically of it and limits scope to the treatment of the ensuing paralyses. Electricity, in its various forms, is dismissed very courteously after a brief synopsis. Exercises, prosthetic appliances, operative measures are discussed in detail and, both in the introductory chapters and as occasion arises in connection with various lesions, diagnostic methods are fully treated.

---

**Studies in Immunization Against Tuberculosis.** Drs. Karl and Silvio von Ruck, Asheville, N. C. Paul B. Hoeber, N. Y. 439 pages, $4.

This work is divided into three parts, Theoretic Considerations, Practical Immunization and Experimental Studies. The name of von Ruck has long been associated with tuberculosis and with a favorable view toward specific treatment. the very thoroughness of the studies included in this book and the conservatism of the authors, makes it difficult to review in few words. Immunization, moreover, is employed both prophylactically and therapeutically, by various technics and even involving very different principles of preparation of what may generally be termed vaccine. The book is, therefore, one for study of a broad subject and by no means a statistic argument by reports of cures, for a given method of therapeutics. We commend it to all real students of the disease but those looking merely for an easy routine treatment with assurance of positive results will meet with disappointment.

---

**Diseases of Occupation and Vocational Hygiene.** Edited by Geo. M. Kober, M. D., LL. D., Washington and Wm. C. Hanson, M. D., Belmont, Mass. 29 contributors. P. Blakiston's Son & Co., Philadelphia. 918 pages, illustrated, $8.

Part 1, deals with metallic and other poisons, infections especially due to occupation, as anthrax and certain infections and infestations depending on occupation as compelling residence in particular climates, dust, lesions of the cardio-vascular-renal apparatus, fatigue and occupational neuroses, conditions of the mouth, nose, throat, eye, ear, and skin; cancer especially with reference to X-rays and radium; electric injuries and shock.

Part 2 views the general subject from the standpoint of etiology and prophylaxis and includes a detailed discussion of special hygienic measures required for a large number of occupations.

Part 3 covers the relation of clinics, statistics, governmental study and legislation to the prevention of occupational diseases and may be considered as mainly sociologic.

An index of authors, 16 pages, gives an idea of the thoroughness of the study for the preparation of this book and the general index not only facilitates reference but suggests a use of the book for more general purposes not indicated by the title. The work, as a whole, is monumental, of wide interest, appalling in its showing of waste of life, life-saving in its ultimate influence.

---

**Fables.** E. P. Anschutz, M. D., published by the Bulletin of the Sons of the Academy. 56 pages, paper cover, 25 cents.

This is a collection of homely truths in fable form, very interesting and none the less instructive.

---

**Practice of Obstetrics,** J. Clifton Edgar, N. Y., published by P. Blakiston's Sons & Co., Philadelphia, 5th revised edition, 22d thousand, 1006 pages, 1316 illustrations, 5 plates and 34 figures in color, $6.00.

This well known work is, by its mere size and abundant illustration, obviously a cyclopaedic monograph. While lucidly written, the illustrations render it almost clinical in presentation of problems and their solution. The summary, preceding each principal division of the work adds to its didactic value by placing before the reader a definite conception of the main subdivisions thus, first giving a bird's-eye view and then bringing him nearer for a detailed consideration of the topics included.

---

**The Practical Medicine Series,** The Year Book Publishers, 327 S. La Salle St., Chicago. Under general editorial charge of Chas. L. Mix, A. M., M. D., 10 volumes a year, $10.

Vol. 4, Series of 1916, Gynaecology, edited by Emilius C.

Dudley, A. M., M. D., and Herbert M. Stone, M. D. 232 pages, $1.35 separately.

Vol. 5, Series of 1916, Paediatrics, edited by I. A. Abt, M. D., and A. Levinson, M. D.; Orthopaedic Surgery, edited by John Ridlon, A. M., M. D., and Chas. A. Parker, M. D., 231 pages, $1.35.

This series is a valuable review of current literature, the offering of each volume separately being an advantage to those whose limitation of interests might prevent subscription to the entire series.

---

**Progressive Medicine.** Edited by H. A. Hare, M. D., and L. F. Appleman, M. D., Philadelphia. Lea & Febiger. Quarterly, paper bound, $6 per annum.

Vol. 19, No. 3, Sept. 1916, 394 pages, is devoted to Diseases of the Thorax, by Wm. Ewart; Dermatology and Syphilis by Wm. S. Gottheil; Obstetrics by E. P. Davis; and Diseases of the Nervous System by Wm. G. Spiller. The volume is well indexed.

